# A PLSPM-Based Test Statistic for Detecting Gene-Gene Co-Association in Genome-Wide Association Study with Case-Control Design

**DOI:** 10.1371/journal.pone.0062129

**Published:** 2013-04-19

**Authors:** Xiaoshuai Zhang, Xiaowei Yang, Zhongshang Yuan, Yanxun Liu, Fangyu Li, Bin Peng, Dianwen Zhu, Jinghua Zhao, Fuzhong Xue

**Affiliations:** 1 Department of Epidemiology and Health Statistics, School of Public Health, Shandong University, Jinan, China; 2 Hunter College - School of Public Health, City University of New York, New York City, New York, United States of America; 3 Bayessoft, Inc., Davis, California, United States of America; 4 School of Public Health, Chongqing Medical University, Chongqing, China; 5 MRC Epidemiology Unit and Institute of Metabolic Science, Cambridge, United Kingdom; Universite de Montreal, Canada

## Abstract

For genome-wide association data analysis, two genes in any pathway, two SNPs in the two linked gene regions respectively or in the two linked exons respectively within one gene are often correlated with each other. We therefore proposed the concept of gene-gene co-association, which refers to the effects not only due to the traditional interaction under nearly independent condition but the correlation between two genes. Furthermore, we constructed a novel statistic for detecting gene-gene co-association based on Partial Least Squares Path Modeling (PLSPM). Through simulation, the relationship between traditional interaction and co-association was highlighted under three different types of co-association. Both simulation and real data analysis demonstrated that the proposed PLSPM-based statistic has better performance than single SNP-based logistic model, PCA-based logistic model, and other gene-based methods.

## Introduction

A Genome-wide Association Study (GWAS) typically tests whether certain SNPs have strong associations with predefined trait or disease by applying statistical methods. Hundreds of GWAS’s for complex human diseases or traits were completed over the last decade. Nonetheless, the genetic variants discovered in GWAS’s account for only a small proportion of the heritability of complex diseases [1], [2]. One possible reason is that most GWAS analysis methods test the SNP-phenotype association individually, which has relatively low power in detecting multiple SNPs with small causal effects [3]. Additionally, in human body, genes tend to work collaboratively, especially within specific pathways or modules that are associated with certain diseases [4]–[6]. Therefore, we suspect that the missing proportion of heritability could be partly due to the ignorance of the joint effect of genes contributing to the disease or trait [3], [7]. Complex diseases often result from multiple genes’ interplays within genetic networks, a general term that we used here to represent all kinds of networks defined on gene level, e.g., biological pathways, gene regulatory networks, and gene modules. The idea of multi-gene effect led to the development of genetic network-based analysis for GWAS [8]–[10].

Network inference is a challenging task and proper methods should be proposed in constructing a priori topological structures for establishing genetic networks that contribute to diseases or traits of interest. A knowledge-based approach is commonly adopted for genetic network construction and inference [11]–[14], but it is still underdeveloped in testing whether significant relationships between any two nodes in such networks exist. Theoretically, this can be solved by testing the joint effect of two genes. Traditionally, to detect gene-gene interaction, a product term is usually added to the logistic regression model 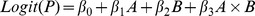, which implies a nearly independence assumption, at least not much correlation, between gene A and gene B for inferring the interaction measurement (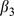) [15], [16]. Nevertheless, one common sense is that the development of most diseases is attributed to the correlated genes in pathways. Another situation is that two SNPs usually locate in the two linked gene regions respectively, or in the two linked exons respectively within one gene. All these situations indicate that the two SNPs may have high correlation rather than independence (or low correlation). Therefore, the assumption of the above logistic model is rarely satisfied, and it will be inevitable to lose efficiency when high correlation existed between the two SNPs. In this paper, we proposed the concept of gene-gene co-association, which refers to the extent to which the joint effects of two genes differs from the main effects, not only due to the traditional interaction under the nearly independent condition but the correlation between two genes, while the part attributed to the correlation has usually been neglected in traditional interaction model using regression method. The proposed gene-gene co-association can be measured by the difference of the correlation between two genes within case and control groups without the independent assumption. This measurement refers to the co-association of two genes contributing to the disease or trait.

For genetic networks derived from GWAS, there are multiple variants (i.e. SNPs) within a gene region, where one single SNP in this region is inadequate to represent the overall effect of the whole gene on a disease. Previous studies suggested that gene-based analysis would allow the formation of pathways to interpret complex diseases and provide the functional bases of an association finding [17]. Therefore, summarizing SNP effects at gene level to estimate gene-gene co-association appears to be an appealing strategy for constructing genetic networks. In our previous study [18], a statistic called CCU for detecting gene-gene co-associations was proposed, which was constructed by the difference between the canonical correlation within case and control respectively. Since CCU statistic only uses the first canonical correlation coefficient, it may not be an inefficient estimator of gene-gene co-associations and may have very low power. Recently, another gene-based statistic was proposed to detect gene-gene interaction [19], which was built based on the difference of the covariance matrix within case and control respectively. Although both the two methods were severely affected by the high multicollinearity problem commonly encountered in GWASs, they motivated us to develop a new gene-based method to detect gene-gene co-association.

In this paper, we proposed a novel statistic to test the co-association between two genes under a case-control design. The statistic was defined as the standardized difference of path coefficient for the gene pair between cases and controls based on Partial Least Squares Path Modeling (PLSPM) [20], [21], which has been successfully used to detect associations in GWAS [22], [23]. To assess the performance of the proposed PLSPM-based statistic, simulation studies were conducted to evaluate its type I error rate and power. Its performance was also compared with single SNP-based logistic regression model [24], [25], Principle Component Analysis(PCA)-based logistic regression model [26], [27], the CCU statistic [18] and the covariance-based statistic [19]. Our method was then applied to real data analysis of Coronary atherosclerotic disease (CAD) association study. Both simulation and real data analysis suggested that the proposed PLSPM-based statistic has advantageous performances compared to other methods.

## Materials and Methods

### The Modeling Framework

**Figure 1:**
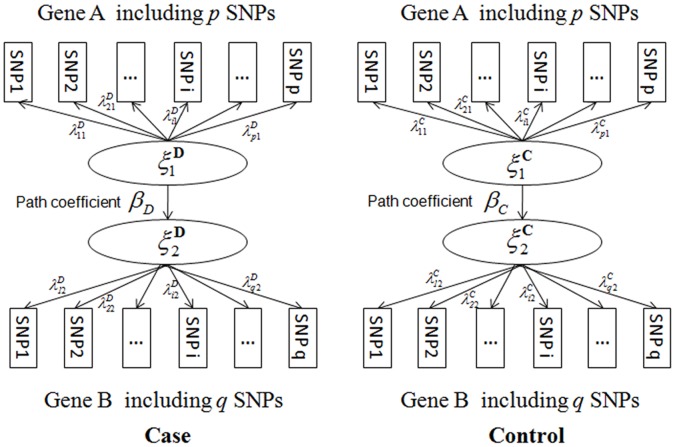
PLSPM-based co-association model.

Figure 1 illustrates the framework for the PLSPM-based statistic between gene A and gene B. We denote the genotype data for gene A and gene B as 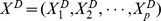 and 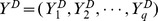 respectively among cases, with_
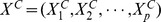
_ and 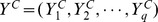 respectively among controls. Then, the path coefficient 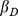 between
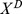
and 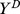
obtained by PLSLM could be viewed as a measure of the correlation between genes A and B among cases. Similarly
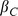
measures the correlation between A and B among controls. In the algorithm of PLSPM, the path coefficient is calculated as the standardized regression coefficient of the two latent variables. This standardized path coefficient is equal to their correlation coefficient between the two latent variables. Therefore the arrow is merely used to reflect the structure and has no direction effect. No matter whether the path coefficients of the two genes are calculated from A to B or from B to A, technically the result remains the same under PLSPM.

We introduce 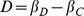 as an estimate of co-association between the two genes contributing to the disease, hence the proposed novel PLSPM-based test statistic can be defined as
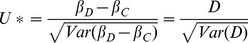
(1)where 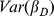,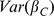,
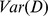
denote the variance of 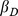,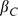, and 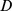 respectively.

The framework of the PLSPM for gene-gene co-association resembles structural equation modeling (SEM) with three types of parameters defined: (1) latent variable scores (i.e., 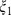 and 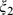) defined as combinations of their manifest variables (SNPs within the gene); (2) path coefficients (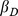 and 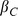) between the two latent variables in the case and control groups, which are counterparts of correlation coefficients in the SEM framework; (3) loadings (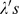) for each block that defines the relationship between the SNPs and their latent variables. In this paper, reflective measurement model was assumed in PLSPM to describe the relationship between SNPs and the latent variables. For estimation of the above parameters, the Lohmöller’s PLSPM algorithm [28] was used. After centering and standardizing the manifest variables (i.e., variables in coding the genotype data such as the additive model) and giving initial values on weights 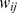
s, the algorithm is essentially an iterative procedure that works by alternating the outer and inner estimation steps. First, in the outer estimation step, we estimate the values of the latent variables 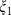 and 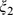 by 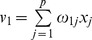 and 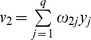, respectively. Then, in the inner estimation step, the endogenous latent variable 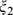 is updated with value 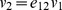, where 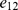
is obtained via the centroid scheme by setting as ‘+1’ or ‘−1’, i.e., the sign of the correlation between the outer estimates 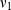 and 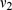. After the inner estimation step, weights are updated before moving to the next step: 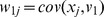 and 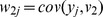. Details of the algorithm and proof of its convergence is similar to the case of the two latent variables as provided in Chapter 2 of the book by Esposito [20]. In GWAS data with case-control design, we separately applied the above algorithm for estimating the path coefficients for cases and for controls.

### Permutation Test for the PLSPM-based Statistic

To test whether genes A and B has co-association effect on a disease of interest, we conduct hypothesis testing with null hypothesis
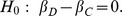



Since PLSPM adopts nonparametric paradigm for estimating 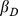 and 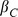 and does not assume parametric distributional forms for the observed and latent variables, the asymptotic distribution of the path coefficients 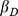 and 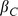 is not available, hence we do not have a distribution available for 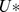
either. To solve this problem, we adopted the strategy of a permutation test [29], [30], a common approach for nonparametric statistical inferences. To alleviate the high computation burden, a random permutation test for 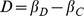 was used to obtain p-value in testing the above 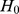. Rejection of the 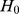 provides evidence in suggesting a significant co-association between the two genes contributing to the disease.

Significance test of path coefficients and loadings were furnished by bootstrap procedures conducted in the case and control groups, respectively [21], [31]. A large, pre-specified number of bootstrap samples (e.g., 1,000), each with the same number of subjects as the original sample, were generated via re-sampling with replacement. Parameter estimation was done for each bootstrap sample, whose path coefficients or loadings can be viewed as drawings from their sampling distributions. All bootstrap samples together provided empirical estimators for the standard error of each parameter.

### Simulation Studies

Simulation studies were conducted to evaluate the performance of the proposed statistic for testing co-association between two genes. We simulated three scenarios by considering different types of co-association: Type I (co-association under nearly independent condition between gene A and gene B), Type II (co-association only caused by correlation between gene A and gene B), and Type III (co-association caused by both correlation and independent term A×B between gene A and gene B).

**Figure 2:**
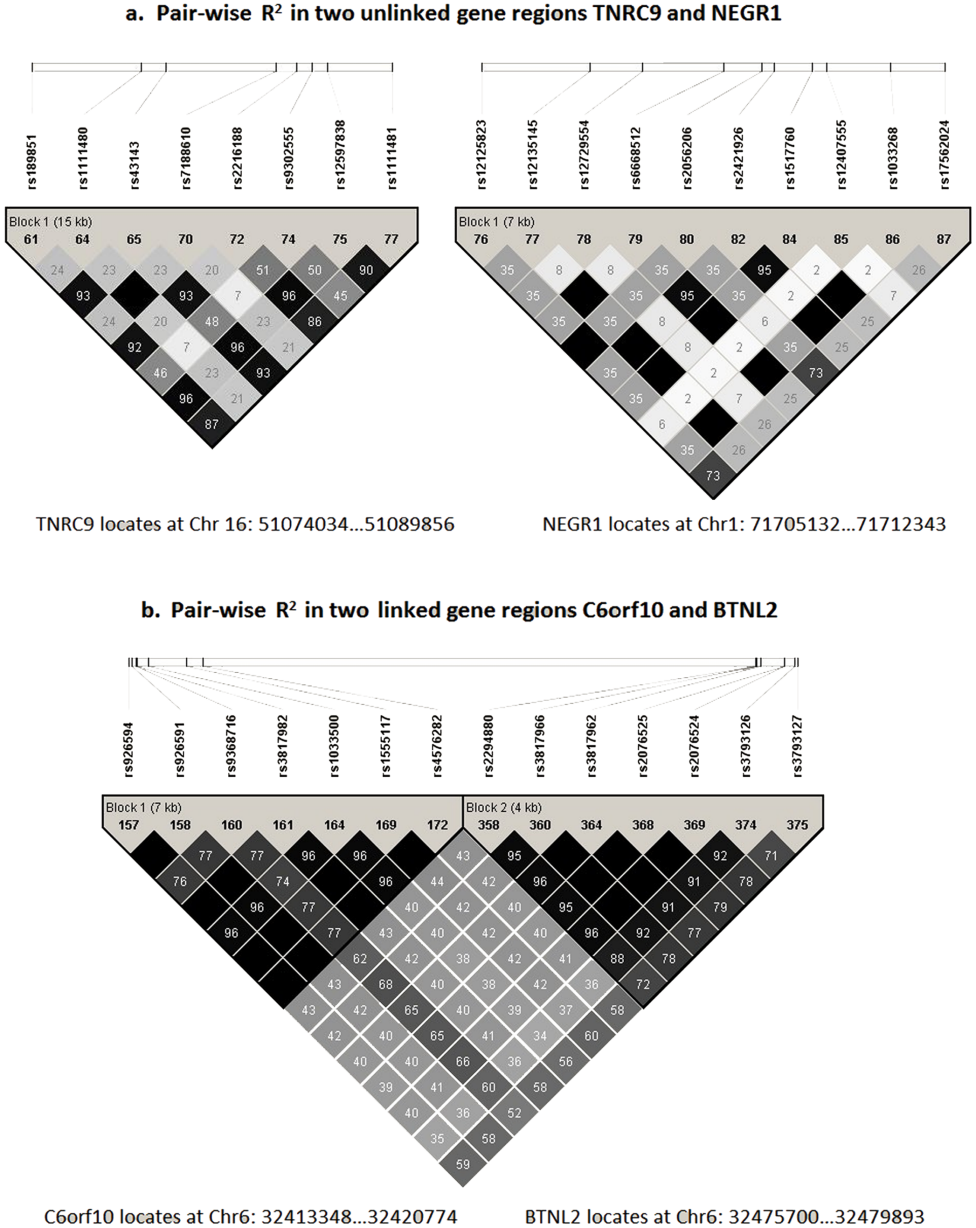
Pair-wise R^2^ in the selected gene regions.

For scenario 1 (Type I co-association), we simulated two causal SNPs with interactions using software gs2.0 [32]. The phased haplotype data of two gene regions TNRC9 and NEGR1 of CEU population were downloaded from the Hapmap website (http://hapmap.ncbi.nlm.nih.gov/) and used to generate the simulation datasets. TNRC9 locates at Chr16∶51074034…51089856, including 8 SNPs, and NEGR1 locates at Chr1∶71705132…71712343, including 10 SNPs. The pair-wise linkage disequilibrium LD pattern of the two gene regions are shown in Figure 2a. For two causal SNPs, SNP1 from gene A and SNP2 from gene B, gs2.0 [32] simulated genotypes and the binary phenotype according to logistic interaction model 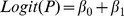
×
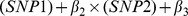
×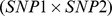, where 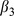 denoted the interaction effect of two SNPs. Furthermore, we specified different interaction odds ratios (ORs, 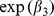) from 1.0 to 1.5 stepped by 0.1.

For scenario 2 (Type II co-association), to create the co-association between linked genes under the condition of none interaction**,** we simulated two linked (correlated) causal SNPs only with marginal effects using software Hapgen2 [33], and further specified co-association levels by the difference of the marginal effects of two causal SNPs. The phased haplotype data of two linked gene regions C6orf10 and BTNL2 of CEU population were downloaded from the Hapmap website and to generate the simulation data. C6orf10 locates at Chr6∶32413348…32420774, including 7 SNPs and BTNL2 locates at Chr6∶32475700…32479893, including 7 SNPs. The pair-wise LD pattern of the two gene regions are shown in Figure 2b. For two causal SNPs, SNP1 from gene A and SNP2 from gene B, Hapgen2 [33] simulated genotypes and the binary phenotype according to logistic model 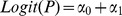
×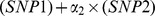. We specified different pairs of marginal effect ORs (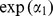,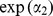) : (1.0, 1.0), (1.5, 1.5), (1.4, 1.6), (1.3, 1.7), (1.2, 1.8) and (1.1, 1.9).

For scenario 3 (Type III co-association), again the same C6orf10 and BTNL2 genes was used in this scenario. Gs2.0 [32] was first used to generate the dataset of Type I co-association, and Hapgen2 [33] for the dataset of Type II co-association. Finally, we mixed the above simulation data with the proportion 1∶1 to create the scenario of Type III co-association. The model can be also expressed by 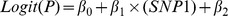
×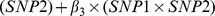, but the two genes are actually correlated rather than independent as defined in the model of scenario 1.

Current GWAS is still map-based rather than sequence-based, so association might predominantly be indirect. We therefore mainly deal with the indirect association. All the datasets were analyzed with the causal SNPs removed, permitting the effect of the causal SNPs to be detected indirectly. The genotype data were coded according to the additive genetic model [25], [34].

Under the null hypothesis 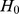 (with 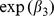 specified as 1.0 in scenario 1 and (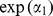,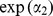) specified as (1.0, 1.0) in scenario 2), 100,000 cases and 100,000 controls were generated and combined to form a hypothetical population from which case and control samples were randomly selected with different sample sizes (N = 1000, 2000, 3000, 4000 or 5000). To examine the stability of the PLSPM-based statistic, we randomly sampled *N* individuals from the cases and controls for the calculation of the type I error rates under different nominal levels of 0.01, 0.05 and 0.1. A total of 1000 simulations were repeated for each sample size.

To highlight the advantages of our proposed PLSPM-based statistic, four existed methods were used to compare with our method. The first was traditional single SNP-based logistic model. For each simulation, all pair-wise SNPs from genes A and B and their product terms were defined as the independent variables in the single SNP-based logistic regression model [24], [25]. We considered each of the pair-wise interactions separately, selecting the most significant one (smallest *p*-value). Significance levels are determined using permutations to adjust the multiple testing. The second was PCA-based logistic model, which was constructed by 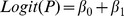
×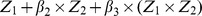, where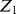 and 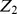 denoted the first principle component score of gene A and gene B respectively, and 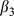 denoted the interaction effect of two genes. The third was the CCU statistic proposed in our previous study, and the last was the recently proposed covariance-based statistic [19].

For scenarios 1 and 2, under the alternative hypothesis
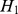
_,_ the performance of four different methods (PLSPM-based statistic, CCU statistic [18], single SNP-based [24], [25] and PCA-based [26], [27] logistic model) were assessed 1) at different sample sizes under fixed OR; 2) at different co-association levels under fixed sample sizes; and 3) at different minor allele frequency (MAF) of causal SNPs from two genes under fixed OR and fixed sample size to evaluate the performance with various linkage disequilibrium (LD) patterns. For scenario 3, under the alternative hypothesis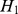, the performance of the four methods were assessed at different sample sizes with fixed co-association level and assessed at different co-association levels with fixed sample sizes. In addition, we compared our PLSPM-based statistic with the covariance-based statistic [19] by repeating 1) and 2) under scenario 1 and 2.

### Application

The proposed PLSPM-based statistic was also applied to a real dataset. The data consisted of genotypes data from three candidate susceptibility genes (LRP5, LRP6, PCSK9), all belonging to the lipid metabolism pathway associated with Coronary atherosclerotic disease (CAD). The dataset contained samples from 498 CAD cases and 509 controls, and the genotyping was conducted by Qilu Hospital of Shandong University in China [35]. The three genes (LRP5, LRP6, PCSK9) were typed with two, nine, three SNPs respectively. All the four methods were conducted in detecting gene-gene co-association contributing to CAD.

## Results

### Simulation Results

#### Type I error rate

Table 1 shows the estimated type I error rates of the PLSPM-based statistic under different nominal levels in both scenario1 and 2. It reveals that the type I error rates of the proposed statistics are close to nominal levels (0.01, 0.05, 0.1) as a function of sample sizes.

**Table 1:** Type I error rates of the PLSPM-based statistic in different scenarios.

	Scenario1			Scenario2		
Sample size	α = 0.01	α = 0.05	α = 0.1	α = 0.01	α = 0.05	α = 0.1
1000	0.017	0.051	0.103	0.013	0.046	0.102
2000	0.011	0.045	0.095	0.011	0.052	0.095
3000	0.010	0.040	0.098	0.012	0.053	0.105
4000	0.012	0.053	0.101	0.010	0.048	0.101
5000	0.011	0.049	0.103	0.015	0.051	0.096

#### Power

**Figure 3:**
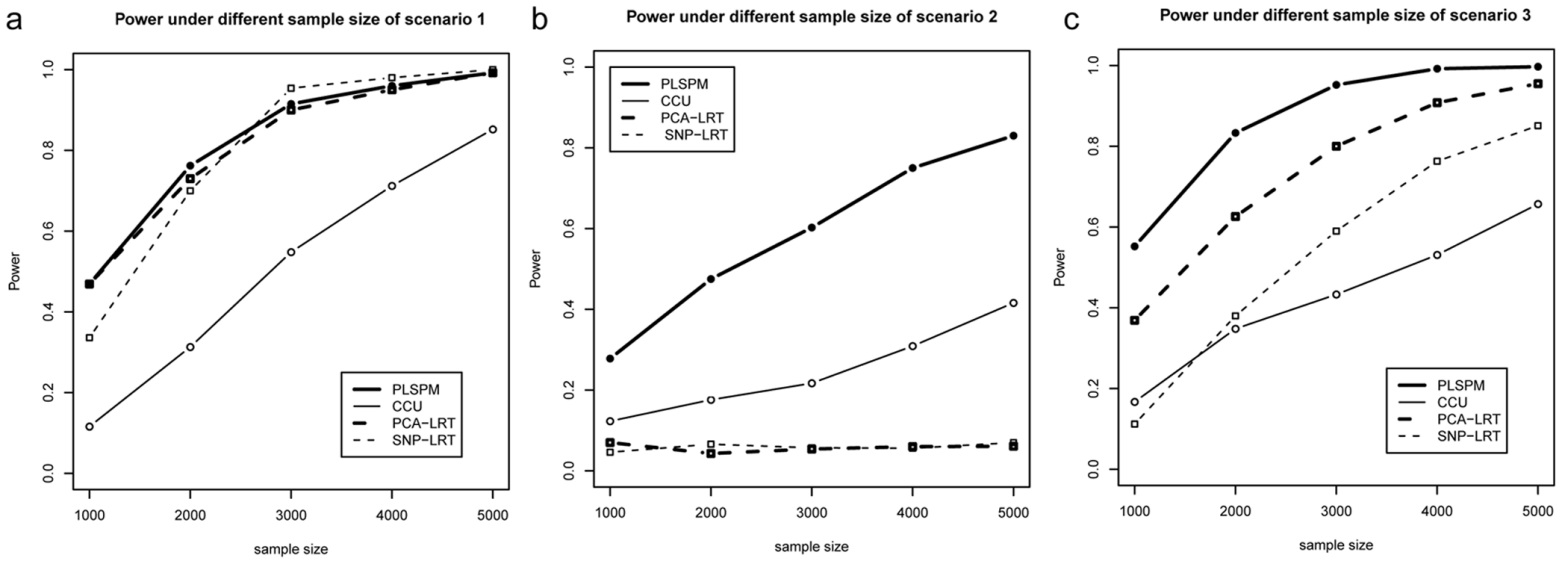
The power of the four methods under different sample sizes. Note: In Figure 3a, rs189851 (MAF = 0.43) in gene TNRC9 and rs12125823(MAF = 0.44) in gene NEGR1 were defined as causal SNPs with their interaction odds ratio fixed at 1.3. In Figure 3b, rs926594 (MAF = 0.46) in gene C6orf10 and rs2294880 (MAF = 0.45) in gene BTNL2 were defined as causal SNPs with their marginal odds ratio fixed at 1.3 and 1.7 respectively. In Figure 3c, mixed dataset with proportion 1∶1 were generated by the same causal SNPs in Figure 3b, with interaction odds ratio 1.3 for Type I co-association and marginal effect odds ratio 1.3 and 1.7 for Type II co-association.

Figure 3 shows the performances of the four methods under different sample sizes given fixed co-association level for scenarios 1, 2 and 3. It indicates that the powers of the four methods all increase monotonically with sample size in scenarios 1 and 3 (Figure 3a, 3c), while the single SNP-based [24], [25] and PCA-based [26], [27] logistic model lost their power in detecting gene-gene Type II co-association (Figure 3b). Obviously, the power of the PLSPM-based statistic is higher than that of the CCU statistic [18]. Only in scenario 1, the single SNP-based logistic model has slight higher power when sample size is larger than 3000, and PCA-based logistic regression model [26], [27] has comparable power with PLSPM-based statistic (Figure 3a), while they has less power for the other two scenarios.

**Figure 4:**
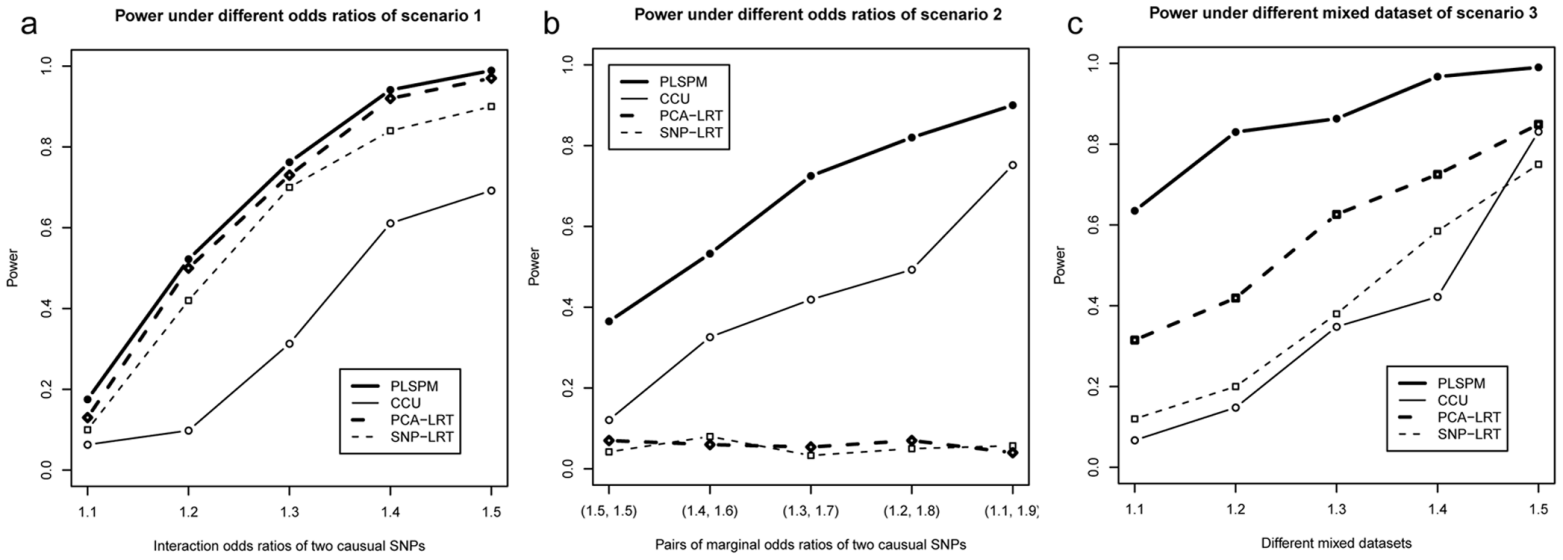
The power of four methods under different co-association levels. Note: In Figure 4a, rs189851 (MAF = 0.43) in gene TNRC9 and rs12125823(MAF = 0.44) in gene NEGR1 were defined as causal SNPs with sample size fixed at 2000. In Figure 4b, rs926594 (MAF = 0.46) in gene C6orf10 and rs2294880 (MAF = 0.45) in gene BTNL2 were defined as causal SNPs with sample size fixed at 4000. In Figure 4c, mixed datasets with proportion 1∶1 were generated by the same causal SNPs in Figure 4b with sample size fixed at 2000, and the horizontal axis denotes different interaction odds ratios for Type I co-association and marginal effect odds ratios for Type II co-association.

Figure 4 depicts the power under different co-association levels in the three scenarios. For the case of Type I co-association in scenario 1, the power increases monotonically with the interaction ORs for all the four methods (Figure 4a). In scenario 2, the power of the PLSPM-based statistic and that of the CCU statistic [18] both increases monotonically along with the difference between marginal ORs of the two causal SNPs (Figure 4b). As for scenario 3, the PLSPM-based statistic has the highest power, followed by the two logistic regression models, and then by the CCU statistic [18].

**Figure 5:**
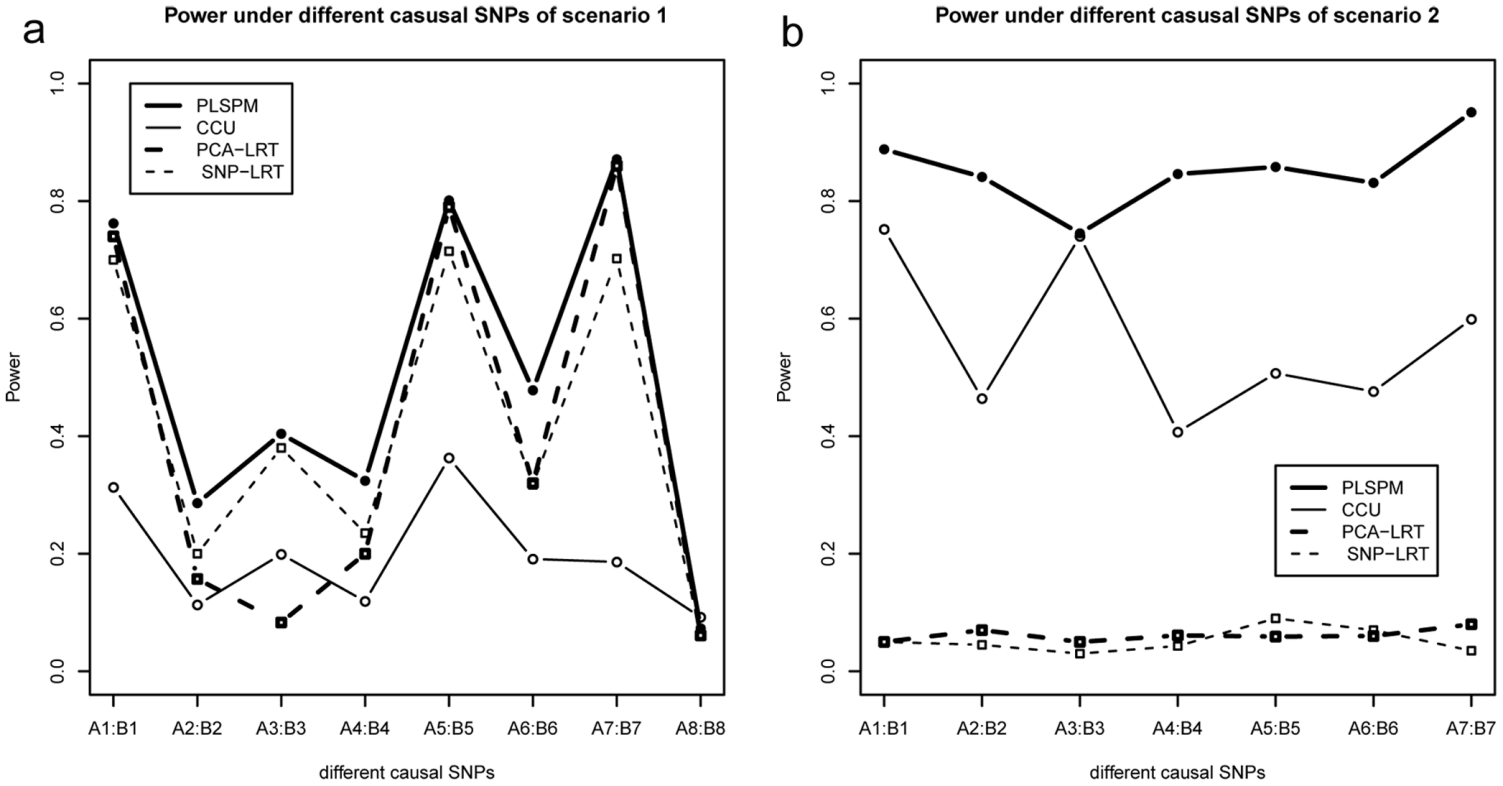
The power of the four methods under different causal SNPs. Note: The horizontal axis denotes the positions of the causal SNPs in the corresponding genes (Ai:Bi denotes the causal SNPs are ith SNP in gene A and ith SNP in gene B). In Figure 5a, A,B denotes gene TNRC9 and NEGR1 with causal SNPs’ interaction odds ratio fixed at 1.3. In Figure 5b, A,B denotes gene C6orf10 and BTNL2 with causal SNPs’ marginal effect odds ratios fixed at 1.3 and 1.7. Results for other pair-wise SNPs are qualitatively similar, hence not shown in the Figure.

Figure 5 illustrates the power of the four methods under different MAF or LD patterns. For both type I and type II co-association, PLSPM-based statistic outperforms all other methods with the highest testing power, although the powers of the four methods vary heavily under different MAF or LD patterns. It is notable that the logistic regression models do not work for scenario 2. Specifically, the power for detecting co-association between the 8th SNP within gene A and 8th SNP gene B is quite low for all the four methods due to the low MAF (0.08) of 8th SNP within gene B (Figure 5a). This indicates that the proposed PLSPM-based statistic lose its power in detecting rare variation.

One reviewer suggested us compare our proposed PLSPM-based statistic with the recently proposed covariance-based statistic [19]. As the covariance-based statistic [19] didn’t work in our simulated data due to that the matrix W defined in their method was not invertible resulted from high collinearity between SNPs, we just attempted to do the calculations using the Moore-Penrose generalized inverse. The results are shown in the Tables S1–S4 in Supplementary Materials S1. In scenario 1, it indicates that the powers of the two methods are comparable in detecting Type I co-association, and the PLSPM-based method has slight advantage with a lower odds ratio which is more common for SNP data. While in scenario 2, the covariance-based statistic [19] has a higher power in detecting the gene-gene Type II co-association.

### Application Result

Table 2 shows the results of a gene-gene co-association test between three genes that are potentially contributing to CAD within the lipid metabolism pathway using the PLSPM-based statistic, CCU statistic [18], single SNP-based logistic model [24], [25] and PCA-based logistic model [26], [27]. The co-association between LRP5 and LRP6 is statistically significant (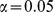) detected only by PLSPM-based statistic and not by the other three methods.

**Table 2:** The results of gene-gene co-association contributing to CAD within the lipid metabolism pathway using four different methods.

	PLSPM-based statistic	CCU	PCA-based logistic model	SNP-based logistic model	
Co-association	P-value	P-value	P-value	SNP-SNP*	P-value
LRP5-LRP6	0.025	0.393	0.275	rs3736228–rs2302685	0.075
LRP5- PCSK9	0.106	0.566	0.681	rs3736228–rs2495477	0.216
LRP6- PCSK9	0.402	0.496	0.503	rs2284396–rs2483205	0.462

* Only the SNP pairs with the smallest P-value were presented.

## Discussion

Many methods have been developed for constructing the genetic network, such as Bayesian network [36], Gaussian network [37], and Boolean network [38]. In these genetic networks for GWAS with case-control design, an ‘edge’ between any two nodes indicates that the joint effects of the two genes on target trait or phenotype would be different between controls and cases, which implies the co-association (or interaction) between the two genes. Various algorithms have been developed to learn the topological structure (i.e., links between the nodes) from GWAS data. In this paper, we proposed a novel statistic within the framework of PLSPM, which can be used to test on the existence of gene-gene co-association, i.e., whether an edge between any two genes would exist. It provides a preliminary or prior tool as a first step in constructing or learning genetic network structures given a GWAS dataset with case-control design.

The concept of gene-gene co-association was proposed in our previous paper [18]. It can be measured by the difference of the gene-gene correlation between the case and control groups without employing the nearly independence (at least not much correlation) assumption. Several strategies could be used to detect the gene-gene co-association, though some of these methods still didn’t jump out of the traditional concept of gene-gene interaction [15], [16]. In this paper, the proposed PLSPM-based statistic clarified the concept and the measurement of gene-gene co-association, which refers to the effects not only due to the traditional interaction under nearly independent condition but the correlation between two genes.

Through simulation, the relationship between traditional interaction and co-association was highlighted. The scope of co-association includes the following three scenarios: co-association under nearly independent condition between gene A and gene B (Figures 3a, 4a, 5a), co-association only caused by correlation between gene A and gene B (Figures 3b, 4b, 5b) and co-association caused by both correlation and independent term A×B between gene A and gene B (Figures 3c, 4c). Currently, simulation and real data analysis demonstrated that the proposed PLSPM-based statistic is stable and has higher power than CCU statistic [18], single SNP-based logistic model [24], [25] and PCA-based logistic model [26], [27] (see results in Table 1, Figure 3 to Figure 5 and Table 2). In addition, the performance of PLSPM-based statistic compared with recently proposed covariance-based statistic [19] indicated that the powers of the two methods are comparable in detecting gene-gene co-association, while the former can deal with the high multicollinearity problem between SNPs (see Supplementary Materials S1).

Observing that two genes in any pathway, two SNPs usually locate in the two linked gene regions respectively or in the two linked exons respectively within one gene are often correlated with each other, we think it is meaningful to fabricate the term, gene-gene co-association. In Peng et al [18], CCU statistic was developed for estimating and testing such a gene-gene co-association within the framework of canonical correlation analysis. Nonetheless, since the CCU statistic [18] was calculated only by the first canonical correlation coefficient, it may lose power in the testing. Our simulation studies confirmed that the novel PLSPM-based statistic had more power than the CCU statistic [18] (see evidence from Figures 3, 4 and 5). Although the power of PLSPM-based statistic is similar as PCA-based logistic model [26], [27] for the case of Type I co-association (Figures 3a, 4a), the former still has a superior performance when the logistic model lose its power for the case of Type II co-association (Figures 3b, 4b, 5b). The logistic regression model methods do not work at all because it cannot theoretically handle the scenario of Type II co-association; PLSPM-based statistic outperforms PCA-based logistic regression model [26], [27] because of the advantage of PLSPM method [20], [21]; PLSPM-based statistic outperforms single SNP-based logistic model [26], [27] since the causal SNPs were excluded and the PLSPM-based statistic reflects the joint effects of multiple SNPs in the genes or regions. Also, the performance of PLSPM-based statistic are comparable with the recently proposed covariance-based statistic [19], while it is not affected by high multicollinearity between SNPs (see Supplementary Materials S1).

The proposed method for detecting gene-gene co-association was developed based on PLSPM. An advantage of the algorithms is that they are robust to the multicollinearity problem, which is commonly encountered in GWAS data because of strong linkage disequilibrium between SNPs [39]–[41]. Compared to covariance-based Structural Equation Model (SEM) and other parametric modeling methods, PLSPM is a “soft modeling” approach, requiring fewer distributional assumptions, and the variables studied can be numerical, ordinal, or nominal, hence no normality assumptions are needed [20]. This is a very appealing feature for SNP data in genetic analysis and PLSPM has been successfully applied in genome wide association studies. We want to admit that although the proposed PLSPM-based approach has indicated numerous benefits, it has some limitations. Firstly, the current PLSPM-based statistic is based on a random permutation test due to the lack of its asymptotic distribution. Parametric test will be in great demand in future studies. Secondly, the PLSPM-based statistic still lacks efficiency when dealing with rare variation situation (see evidence in Figure 5a).

## Supporting Information

Supplementary Materials S1 Table S1. The power of the two methods for detecting Type I co-association under different sample sizes. Table S2. The power of the two methods for detecting Type I co-association under different interaction odds ratios. Table S3. The power of the two methods for detecting Type II co-association under different sample sizes. Table S4. The power of the two methods for detecting Type II co-association under different pairs of marginal odds ratios.
(DOC)

## References

[1] Visscher PM (2008) Sizing up human height variation. Nature Genetics 40: 489–490. 18443579 10.1038/ng0508-489

[2] Stranger BE, Stahl EA, Raj T. (2011) Progress and promise of genome-wide association studies for human complex trait genetics. Genetics: 187(2), 367–403. 10.1534/genetics.110.120907 PMC3030483 21115973

[3] Manolio TA, Collins FS, Cox NJ, Goldstein DB, Hindorff LA, et al (2009) Finding the missing heritability of complex diseases. Nature 461: 747–753. 19812666 10.1038/nature08494 PMC2831613

[4] Barabási AL, Oltvai ZN (2004) Network biology: understanding the cell’s functional organization. Nature Reviews Genetics 5: 101–113. 10.1038/nrg1272 14735121

[5] Oti M, Brunner HG (2007) The modular nature of genetic diseases. Clinical genetics 71: 1–11. 17204041 10.1111/j.1399-0004.2006.00708.x

[6] Li Y, Agarwal P (2009) A pathway-based view of human diseases and disease relationships. PLoS One 4: e4346. 19194489 10.1371/journal.pone.0004346 PMC2631151

[7] Frazer KA, Murray SS, Schork NJ, Topol EJ (2009) Human genetic variation and its contribution to complex traits. Nature Reviews Genetics 10: 241–251. 10.1038/nrg2554 19293820

[8] Torkamani A, Schork NJ (2009) Pathway and network analysis with high-density allelic association data. Methods Mol Biol 563: 289–301. 19597792 10.1007/978-1-60761-175-2_16

[9] Baranzini SE, Galwey NW, Wang J, Khankhanian P, Lindberg R, et al (2009) Pathway and network-based analysis of genome-wide association studies in multiple sclerosis. Human molecular genetics 18: 2078–2090. 19286671 10.1093/hmg/ddp120 PMC2678928

[10] Jia P, Wang L, Meltzer HY, Zhao Z (2010) Common variants conferring risk of schizophrenia: a pathway analysis of GWAS data. Schizophrenia research 122: 40–42. 10.1016/j.schres.2010.07.001 PMC2933424 20659789

[11] Aerts S, Lambrechts D, Maity S, Van Loo P, Coessens B, et al (2006) Gene prioritization through genomic data fusion. Nature biotechnology 24: 537–544. 10.1038/nbt1203 16680138

[12] Hutz JE, Kraja AT, McLeod HL, Province MA (2008) CANDID: a flexible method for prioritizing candidate genes for complex human traits. Genetic epidemiology 32: 779–790. 18613097 10.1002/gepi.20346 PMC4420475

[13] Bush WS, McCauley JL, DeJager PL, Dudek SM, Hafler DA, et al (2011) A knowledge-driven interaction analysis reveals potential neurodegenerative mechanism of multiple sclerosis susceptibility. Genes and immunity 12: 335–340. 21346779 10.1038/gene.2011.3 PMC3136581

[14] Ma L, Brautbar A, Boerwinkle E, Sing CF, Clark AG, et al (2012) Knowledge-Driven Analysis Identifies a Gene–Gene Interaction Affecting High-Density Lipoprotein Cholesterol Levels in Multi-Ethnic Populations. PLoS Genetics 8: e1002714. 22654671 10.1371/journal.pgen.1002714 PMC3359971

[15] Miettinen O (1974) Confounding and effect-modification. Am J Epidemiol 100: 350–353. 4423258 10.1093/oxfordjournals.aje.a112044

[16] Ahlbom A, Alfredsson L (2005) Interaction: A word with two meanings creates confusion. Eur J Epidemiol 20: 563–564. 16119427 10.1007/s10654-005-4410-4

[17] Luo L, Peng G, Zhu Y, Dong H, Amos CI, et al (2010) Genome-wide gene and pathway analysis. European Journal of Human Genetics 18: 1045–1053. 20442747 10.1038/ejhg.2010.62 PMC2924916

[18] Peng Q, Zhao J, Xue F (2009) A gene-based method for detecting gene–gene co-association in a case–control association study. European Journal of Human Genetics 18: 582–587. 20029457 10.1038/ejhg.2009.223 PMC2987308

[19] Rajapakse I, Perlman MD, Martin PJ, Hansen JA, Kooperberg C (2012) Multivariate Detection of Gene-Gene Interactions. Genetic Epidemiology. 10.1002/gepi.21656 PMC3556521 22782518

[20] Esposito VV, Chin WW, Henseler J, Wang H (2010) Handbook of Partial Least Squares: Concepts, Methdos and Applications. Berlin Heidelberg: Springer.

[21] Tenenhaus M, Vinzi VE, Chatelin YM, Lauro C (2005) PLS path modeling. Computational Statistics & Data Analysis 48: 159–205.

[22] Turkmen AS, Lin S. (2011) Gene-based partial least-squares approaches for detecting rare variant associations with complex traits. BioMed Central Ltd. S19. 10.1186/1753-6561-5-S9-S19 PMC3287853 22373126

[23] Xue F, Li S, Luan J, Yuan Z, Luben RN, et al (2012) A Latent Variable Partial Least Squares Path Modeling Approach to Regional Association and Polygenic Effect with Applications to a Human Obesity Study. PloS one 7: e31927. 22384102 10.1371/journal.pone.0031927 PMC3288051

[24] Schlesselman JJ (1982) Case-Control Studies: Design, Conduct, Analysis. Oxford University Press.

[25] Marchini J, Donnelly P, Cardon LR (2005) Genome-wide strategies for detecting multiple loci that influence complex diseases. Nature genetics 37(4): 413–417. 15793588 10.1038/ng1537

[26] Wang K, Abbott D (2008) A principal components regression approach to multilocus genetic association studies. Genet Epidemiol 32(2): 108–118. 17849491 10.1002/gepi.20266

[27] Gauderman WJ, Murcray C, Gilliland F, Conti DV (2007) Testing association between disease and multiple SNPs in a candidate gene. Genet Epidemiol 31(5): 403–395. 10.1002/gepi.20219 17410554

[28] Lohmöller JB (1989) Latent variable path modeling with partial least squares: Physica-Verlag Heidelberg.

[29] Doerge RW, Churchill GA (1996) Permutation tests for multiple loci affecting a quantitative character. Genetics 142: 285. 8770605 10.1093/genetics/142.1.285 PMC1206957

[30] Good PI (2000) Permutation tests: Wiley Online Library, 5–21.

[31] Efron B (1979) Bootstrap methods: another look at the jackknife. The annals of Statistics 7: 1–26.

[32] Li J, Chen Y (2008) Generating samples for association studies based on HapMap data. BMC Bioinformatics 9: 44. 18218094 10.1186/1471-2105-9-44 PMC2375120

[33] Su Z, Marchini J, Donnelly P (2011) HAPGEN2: simulation of multiple disease SNPs. Bioinformatics 27(16): 2304–2305. 21653516 10.1093/bioinformatics/btr341 PMC3150040

[34] Li WT, Reich J (2000) A complete enumeration and classification of two-locus disease models. Hum Hered 50: 334–349. 10899752 10.1159/000022939

[35] Chen MZ, Cheng GH, Ma L, Wang H, Qiu RF, et al (2011) Association study between TNFSF4 and coronary heart disease. Yi chuan = Hereditas/Zhongguo yi chuan xue hui bian ji 33: 239. 10.3724/sp.j.1005.2011.00239 21402531

[36] Han B, Chen X (2011) bNEAT: a Bayesian network method for detecting epistatic interactions in genome-wide association studies. BMC genomics 12: S9. 10.1186/1471-2164-12-S2-S9 PMC3194240 21989368

[37] Jen-hwa C, Scott W, Vincent C, Benjamin R (2009) A graphical model approach for inferring large-scale networks integrating gene expression and genetic polymorphism. BMC Systems Biology 3: 55. 19473523 10.1186/1752-0509-3-55 PMC2694152

[38] Saithong T, Bumee S, Liamwirat C, Meechai A (2012) Analysis and Practical Guideline of Constraint-Based Boolean Method in Genetic Network Inference. PLoS ONE 7: e30232. 22272315 10.1371/journal.pone.0030232 PMC3260258

[39] Fridley BL, Biernacka JM (2011) Gene set analysis of SNP data: benefits, challenges, and future directions. European Journal of Human Genetics 19: 837–843. 21487444 10.1038/ejhg.2011.57 PMC3172936

[40] Reich DE, Cargill M, Bolk S, Ireland J, Sabeti PC, et al (2001) Linkage disequilibrium in the human genome. Nature 411: 199–204. 11346797 10.1038/35075590

[41] Weiss KM, Clark AG (2002) Linkage disequilibrium and the mapping of complex human traits. TRENDS in Genetics 18: 19–24. 11750696 10.1016/s0168-9525(01)02550-1

