# Supplementary material for: A PLSPM-Based Test Statistic for Detecting Gene-Gene Co-Association in Genome-Wide Association Study with Case-Control Design
Source: PLoS One. 2013 Apr 19;8(4):e62129. doi: 10.1371/journal.pone.0062129 (PMC3631168; doi:10.1371/journal.pone.0062129)
Supplement: Supplementary Materials S1 — Table S1. The power of the two methods for detecting Type I co-association under different sample sizes. Table S2. The power of the two methods for detecting Type I co-association under different interaction odds ratios. Table S3. The power of the two methods for detecting Type II co-association under different sample sizes. Table S4. The power of the two methods for detecting Type II co-association under different pairs of marginal odds ratios. (DOC) [file pone.0062129.s001.doc]

Supplementary materials for

“A PLSPM-based test statistic for detecting gene-gene co-association in genome-wide association study with case-control design”

Xiaoshuai Zhang^1^, Xiaowei Yang^2,3^, Zhongshang Yuan^1^, Yanxun Liu^1^, Fangyu Li^1^, Bin Peng^4^, Dianwen Zhu^2^, Jinghua Zhao^5^, Fuzhong Xue^1^*

^1^Department of Epidemiology and Health Statistics, School of Public Health, Shandong University, Jinan, China

^2^Hunter College - School of Public Health, City University of New York, New York City, New York, United States of America

^3^Bayessoft, Inc., Davis, California, United States of America

^4^School of Public Health, Chongqing Medical University, Chongqing, China

^5^MRC Epidemiology Unit and Institute of Metabolic Science, Cambridge, United Kingdom

*Corresponding author: Fuzhong Xue

Postal address: Department of Epidemiology and Health Statistics, School of Public Health, Shandong University, PO Box 100, Jinan 250012, China.

Phone number: +86 13906405997

Email address: xuefzh@sdu.edu.cn

**Supplementary Materials S1**

As one reviewer suggested, we compared our proposed PLSPM-based statistic with the recently proposed covariance-based statistic [19]. Since the covariance-based statistic [19] didn’t work in our simulated data due to that the matrix W defined in their method was not invertible resulted from high collinearity between SNPs, we attempted to do the calculation using the Moore-Penrose generalized inverse. The comparison results are shown in the following Table S1-S4.

**Table S1** The power of the two methods for detecting Type I co-association under different sample sizes

| **Scenario 1** | **1000** | **2000** | **3000** | **4000** | **5000** |
| --- | --- | --- | --- | --- | --- |
| PLSPM-based | 0.469 | 0.762, | 0.915 | 0.96 | 0.992 |
| Covariance-based | 0.322 | 0.762 | 0.925 | 0.989 | 0.999 |

**Table S2 The power of the two methods for detecting Type I co-association under different interaction odds ratios**

| **Scenario 1** | **1.1** | **1.2** | **1.3** | **1.4** | **1.5** |
| --- | --- | --- | --- | --- | --- |
| PLSPM-based | 0.175 | 0.522 | 0.762 | 0.941 | 0.989 |
| Covariance-based | 0.109 | 0.304 | 0.752 | 0.957 | 0.985 |

**Table S3** The power of the two methods for detecting Type II co-association under different sample sizes

| **Scenario 2** | **1000** | **2000** | **3000** | **4000** | **5000** |
| --- | --- | --- | --- | --- | --- |
| PLSPM-based | 0.345 | 0.63 | 0.75 | 0.9 | 0.945 |
| Covariance-based | 0.386 | 0.644 | 0.82 | 0.957 | 0.984 |

**Table S4 The power of the two methods for detecting Type II co-association under different pairs of marginal odds ratios**

| **Scenario 2** | **(1.5,1.5)** | **(1.4,1.6)** | **(1.3,1.7)** | **(1.2,1.8)** | **(1.1,1.9)** |
| --- | --- | --- | --- | --- | --- |
| PLSPM-based | 0.365 | 0.533 | 0.725 | 0.82 | 0.9 |
| Covariance-based | 0.218 | 0.708 | 0.957 | 0.997 | 1 |
